# On the Utility of Botany

**Published:** 1804-04-01

**Authors:** 


### *To the Editors of the Medical and Phyfical Journal.*

GENTLEMEN,

AT a time when the study of Botany has become a source of fashionable and popular amusement, and when the science of medicine, diffusing itself more extensively than at any former period in the public notice, has more or less occupied the attention of the unlearned, it may perhaps be an acceptable and not altogether useless attempt to unite in a popular form, these two branches of science. They are by nature intimately connected; and the powerful and varied effects, which are produced on the human frame by the numerous members of the vegetable kingdom, mark the destined union of the two sciences, and declare, that without the assistance of Botany, the study of Medicine cannot be complete. Chemistry indeed is also an indispensable handmaid to medicine; and, as poetry, painting, and music have been termed the sister arts, so these three may be called the sister sciences. But as chemistry is neither reducible to so precise limits as her sister, nor capable of being brought so much within reach of universal observation, the compiler of the following sheets has confined himself to an enumeration of the benefits, which from botany alone may be derived to the practice of medicine. Where any plant has appeared to be otherwise useful, either in the cultivation of the arts or the improvement of manufactures, as increasing the catalogue of esculents, answering the purposes of domestic economy, or contributing to the advantage of the farmer; these various properties have been carefully collected. The compiler claims no other merit than that of accuracy. He has consulted some of the most approved medico-botanical authors, that have fallen in his way; as well those who professedly treat of the subject in hand, as those who have only glanced at it incidentally in the course of their writings.

While there shall continue to exist a numerous class of society, reduced to the necessity of labour for their daily sustenance, and as long as there shall be found large portions of the world remote from medical assistance, it is impossible that in all cases the care of a legitimate son of Esculapius can be obtained by the sick; and, notwithstanding standing the well intended and, in some degree, just caution given by the learned, zealous, and benevolent high priest of Hygeia, in the second number of his monthly sacrifice at the shrine of that goddess, recourse must often be had for medical advice by the indigent and the solitary, to persons not regularly initiated into the mysteries of the faculty, nor stamped with an M. D. In spite of doctors, diseases will intrude; and it would probably be a task of difficulty to persuade the charitable 'squire's lady, or the humane country clergyman, that true charity and humanity require them to remain strictly neutral during the struggles of a poor helpless neighbour against the assaults of sickness, because they are not licensed by a diploma from Edinburgh or Oxford.

The stores of Nature for the relief of her creatures are unbounded; the disorders of the poor, and those untainted with the corruptions of luxury, are, generally speaking, simple; and every field, every wood, every despised ditch, every barren waste almost that displays itself before the eye of ungrateful man, presents him with a variety of remedies for his sufferings, and of palliatives for the consequences of his own folly or wickedness. To offer these to view in the most popular and comprehensive form is the present design: The compiler has extracted from a considerable number of volumes, and endeavoured to reduce into the smallest compass possible, the various properties ascribed to the indigenous plants of the British Isles: And as far as the knowledge of them appeared to be generally conducive to the advantage of society, or negatively desirable by way of caution against the effects of deleterious vegetables, no pains have been spared to render the detail full and satisfactory.

The botanical department is built chiefly on Dr. Withering's *Bot. Arrangement*; and from thence the descriptions of the plants have been uniformly adopted, as given in his third edition, 1796.

Dr. Woodville's medico-botany is the ground work of that part which relates to medicine; and whatever facts are adduced or opinions recorded, they are each carefully ascribed to their respective authors. In some instances, where quotations have been copied from an intermediate writer, the name of the original author only, has, for the sake of brevity, been cited in this compilation.

*Catalogue of such British Plants as have been found in any Shape serviceable to Man, whether in a medicinal, œconomical, culinary, or agricultural Point of View, together with an Account of the Uses which they have been made to answer, and an accurate botanical Description of each Plant.*

### CLASS I. MONANDRIA.

Monandria, Monogynia.

1. SALICORNIA, *S. herbacea, S. europæa herbacea, S. internodiis longioribus.*

*Anglice.* Jointed glass wort, salt wort, sea-grass, marsh samphire.

*Generic description. Calyx,* rather bellying. *Seed* 1.

*Specific description.* Herbaceous, wide spreading, joints flatted at top and notched, scarcely nine inches long. Flowers in a spike, near together, three on each side. *On the sea shore, common.* BLOSS. Aug. Sept.

*Use.* It is from the ashes of this plant that is obtained a fossil alkali, in great request for making soap and glass, which is known by the name of *soda,* and is chiefly made on the coasts of the Mediterranean. On the coasts of England, the green plant steeped in salted vinegar, is sold as samphire, to which, made into a pickle, it is very little inferior. The whole plant has a saltish taste, and is greedily devoured by cattle. *Withering.*

2. HIPPURIS. *H. vulgaris.*

*Ang.* Mare's-tail, paddow pipe.

*Gen. desc. Cal.* none; *summit,* simple; seed. 1.

*Spec. Desc. Leaves* awl-shaped, eight to twelve in a whirl round the joints, narrow; *stem* straight, jointed; *flowers* equal in number to the leaves, and at the base of each leaf. *Muddy ponds and ditches (not common) in Scotland, Gloucestershire, and N. W. of Lancashire.* BLOS. May.

*Use.* A very weak astringent. Goats eat it, but cows, sheep, horses, and swine refuse it.

### CLASS II. DIANDRIA.

Diandria, Monogynia.

1. LIGUSTRUM. *S. vulgare. S. germanicum.*

*Ang.* Privet. prim. print.

*Gen. desc. Bloss.* four-cleft; *berry,* with two cells, two seeds in each cell.

*Sp. desc. Leaves* egg-spear shaped, entire, in opposite pairs, sometimes sometimes by threes, sometimes alternate. *Blossoms* white; segments thick and fleshy; stamens sometimes three or four. *Berries* black, egg-shaped; continue through the winter. *In hedges on gravelly soils.* Bloss. June, July.

*Use.* The leaves are bitter and slightly astringent. The berries are filled with a dry, spongy, violet pulp, from which a rose-coloured pigment may be prepared. With the addition of alum, the berries dye wool and silk of a good and durable *green*: for this purpose they must be gathered as soon as they are ripe. Oxen, goats, and sheep eat it: horses refuse it.

2. Veronica. *V. officinalis. V. mas supina. V. vera et major.*

*Ang.* Speedwell, male speedwell, fluellin.

*Gen. Desc. Bloss.* border four-cleft; lower segment narrowest; *caps.* two celled.

*Spec. Desc. Leaves* egg-shaped, serrated, hairy underneath and at the edges, opposite. *Stem* trailing. *Floral leaves,* strap shaped. *Blossom,* purplish blue; tube about half as long as the cup, white fruit stalks very near the end of the stem, but not terminating. *On barren ground and heaths.* Bloss. May, Aug.

*Use.* The leaves are slightly astringent and bitter: It is called by the French *the d'Europe,* and Hoffman has, with many others about a century ago, recommended an infusion of them as a substitute for tea, but it is more astringent and less grateful. *With.* It is a good *antiscorbutic,* and has been used in the *gout* and *rheumatism*—*Hill.* As a medicine also numerous virtues have been ascribed to it, especially in disorders of the lungs, as *coughs, asthmas, consumptions,* &c. in which it is said not only to prove *expectorant,* by also by its extraordinary vulnerary power to heal internal ulcers. But to be judged of, by its sensible qualities, it is only to be recognized as an *astringent,* and not being sufficiently powerful to produce any considerable effect, is now disregarded by medical practitioners. *Woodville.* It is eaten by cows, sheep, goats, and horses, but swine refuse it.

3. Veronica. *V. Beccabunga. Anagallis aquatica vulg.*

*Ang.* Brooklime.

*Gen. Desc.* As above.

*Spec. Desc. Leaves* egg-shaped, flat, serrated with glands. *Stem* creeping. *Blossom* blue; bunches lateral; germen sitting on a thick yellowish-green glandular substance *In slow shallow streams,* and near springs that seldom freeze.

freeze. The whole plant smooth and succulent. Bloss. June.

*Use.* This plant was formerly esteemed as an external application to *wounds and ulcers,* and was used in many diseases: it is now valued only for its antiscorbutic virtues; as a mild refrigerant juice, it is preferred in that disease which has been called the hot scurvy. To derive much advantage from it, the juice ought to be used in large quantities, or the fresh plant eaten as food—*Woodville.* The leaves are mild and succulent, and are eaten in sallads early in the spring. Cows, goats, and horses eat it; it is refused by swine.

4. Veronica. *V. chamædrys. Chamædrys spuria. C. sylvestris.*

*Ang.* Wild germander, germander speedwell.

*Gen. Desc.* As above.

*Spec. Desc. Leaves* egg-shaped, sitting, wrinkled, toothed. *Stem* with two opposite rows of hairs. *Bunches* lateral, frequently opposite. *Blossom* fine blue. In pastures, sides of hedges. Blos. May.

*Use.* The leaves are a better substitute for tea than those of the V. officinalis, being more grateful and less astringent. Cows and goats eat it; sheep, horses, and swine refuse it.

[ To be continued. ]

